# Supplementary material for: Selection and co-selection of antibiotic resistances among Escherichia coli by antibiotic use in primary care: An ecological analysis
Source: PLoS One. 2019 Jun 10;14(6):e0218134. doi: 10.1371/journal.pone.0218134 (PMC6557515; doi:10.1371/journal.pone.0218134)
Supplement: S1 File — Figs A-G. Variation in antibiotic prescribing and proportion of urinary samples with E. coli resistant to antibiotics. (DOCX) [file pone.0218134.s001.docx]

**S1. Supplementary material**

**Variation in antibiotic prescribing**

Figs A and B show the variation in antibiotic prescribing over time in Clinical Commissioning Groups (CCGs) in England. There was generally more variation between CCGs than variation over time within CCGs. However, for some antibiotics there were clear peaks in the amount of dispensed antibiotics. Penicillins with extended spectrum (mainly amoxicillin) are mainly prescribed for respiratory tract infections and prescribing clearly peaked during the winter when the incidence of such infections is higher (peak in December, Fig A). β-lactamase-resistant penicillins (mainly flucloxacillin) are mainly prescribed for skin conditions and peaked in July in line with the frequently observed summer peak in the incidence of (Gram-positive) skin infections. β-lactamase-sensitive penicillins (mainly penicillin V) are mainly prescribed for sore throat and peaked in March.

**Fig A. Variation in antibiotic prescribing.**

Data shown for dispensing of tetracyclines, penicillins with extended spectrum, Beta-lactamase-resistant penicillins and macrolide. Each line represents a different clinical commissioning group in England.

**Fig B. Variation in antibiotic prescribing.**

Data shown for dispensing of trimethoprim, nitrofurantoin, beta-lactamase-sensitive penicillins, and other antibiotics. Each line represents a different clinical commissioning group in England.

**Variation in antibiotic resistances**

The Figs (C-G) below show the proportion of *E.coli* urinary samples that is resistant to the antibiotics of interest. The unit of analysis is the Clinical Commissioning Group (CCG). Each month (x-axis) of the boxplot shows the variation across all CCGs, provided that there were sufficient samples tested in that month for the resistance of interest. The Figs show that there is substantial variation between the CCGs (within each month), but that the median (and IQR) proportion of samples being tested as resistant to the antibiotics of interest is more stable over time (between months).


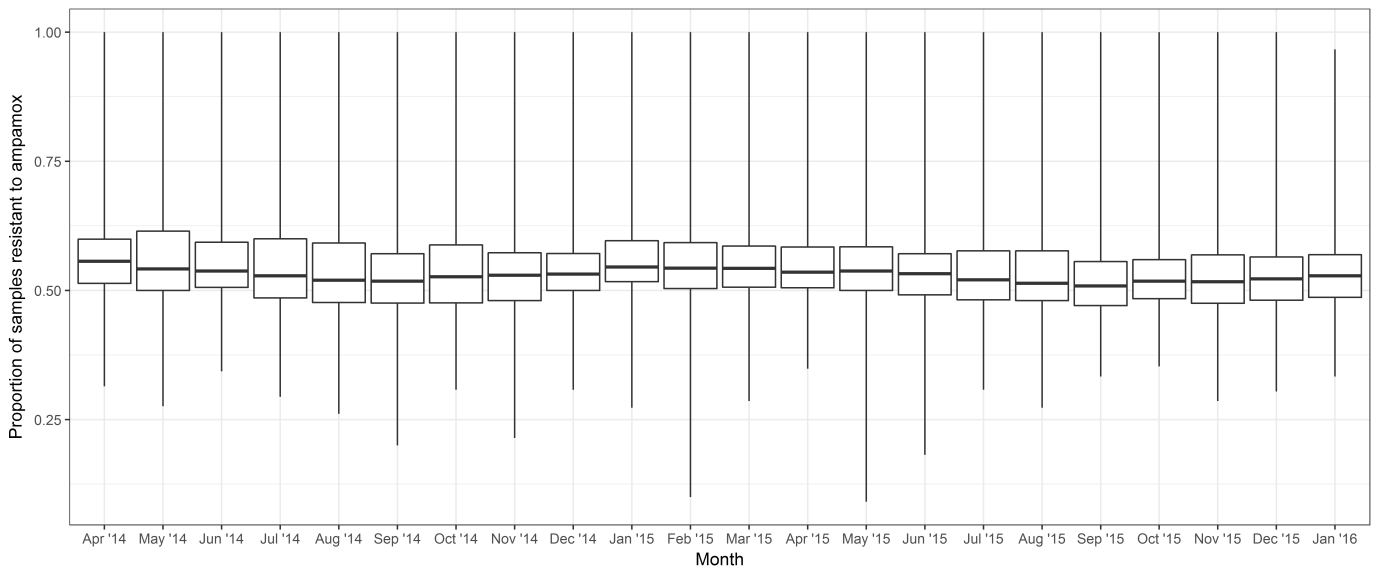


**Fig C. Proportion of urinary samples with E. coli isolated resistant to amoxicillin/ampicillin.**

The boxplot shows the variation in amoxicillin/ampicillin resistance between Clinical Commissioning Groups over time.
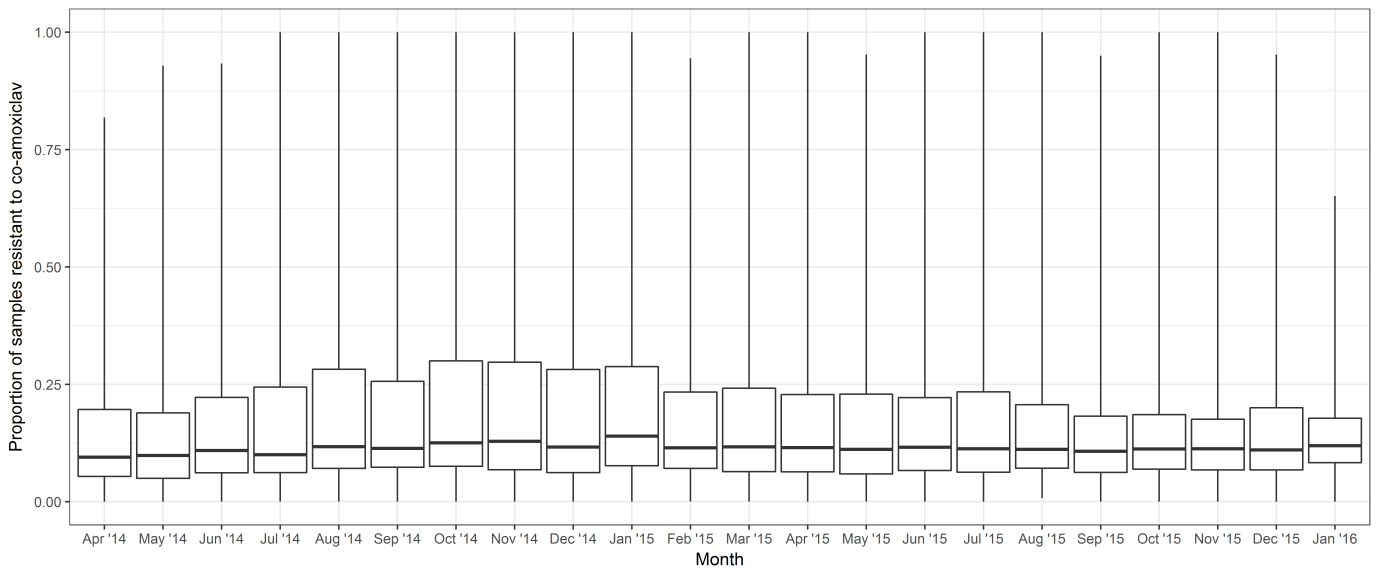


**Fig D. Proportion of urinary samples with E. coli isolated resistant to co-amoxiclav.**

The boxplot shows the variation in co-amoxiclav resistance between Clinical Commissioning Groups over time.


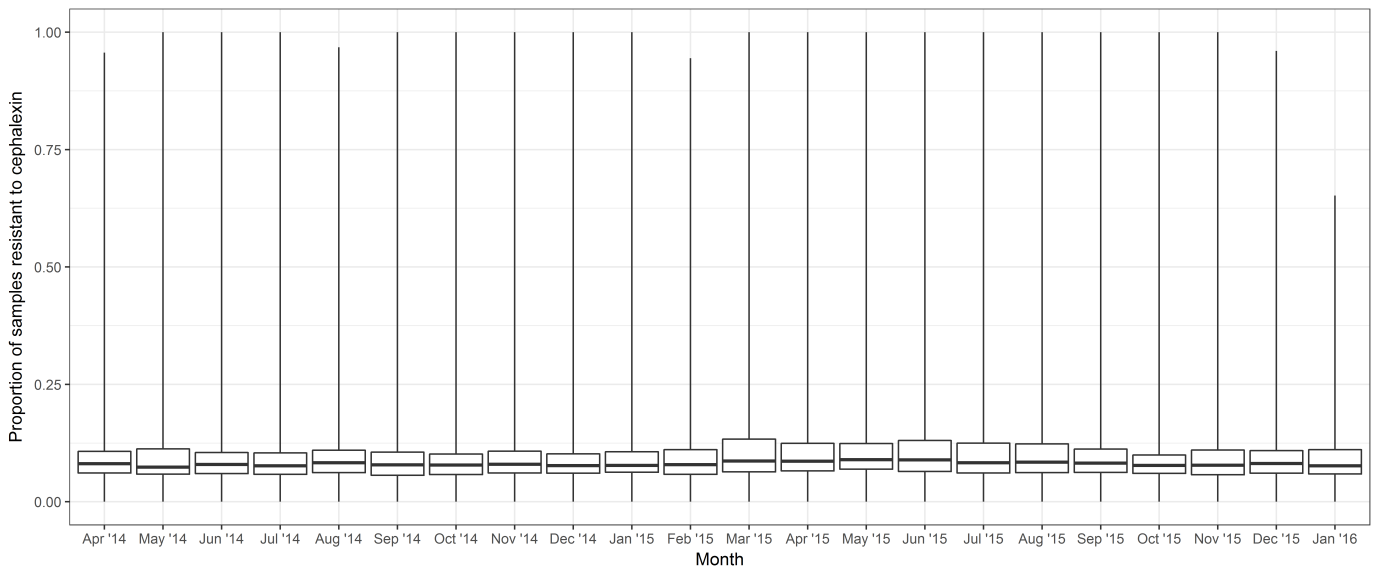


**Fig E. Proportion of urinary samples with E. coli isolated resistant to cephalexin.**

The boxplot shows the variation in cephalexin resistance between Clinical Commissioning Groups over time.


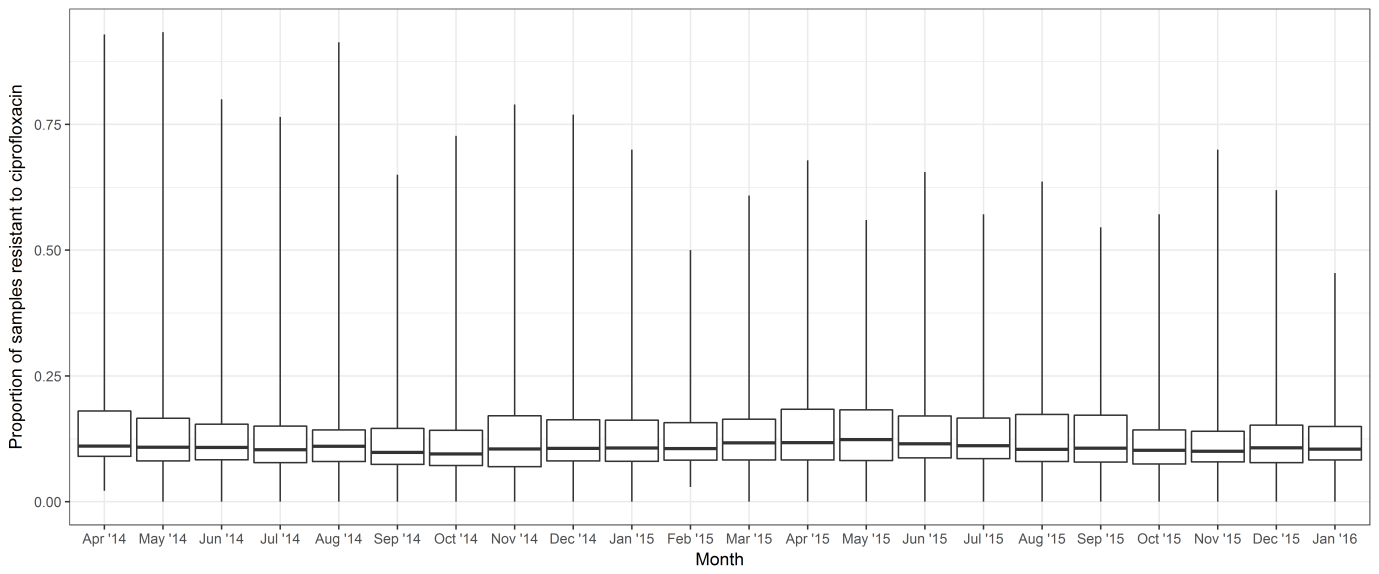


**Fig F. Proportion of urinary samples with E. coli isolated resistant to ciprofloxacin.**

The boxplot shows the variation in ciprofloxacin resistance between Clinical Commissioning Groups over time.


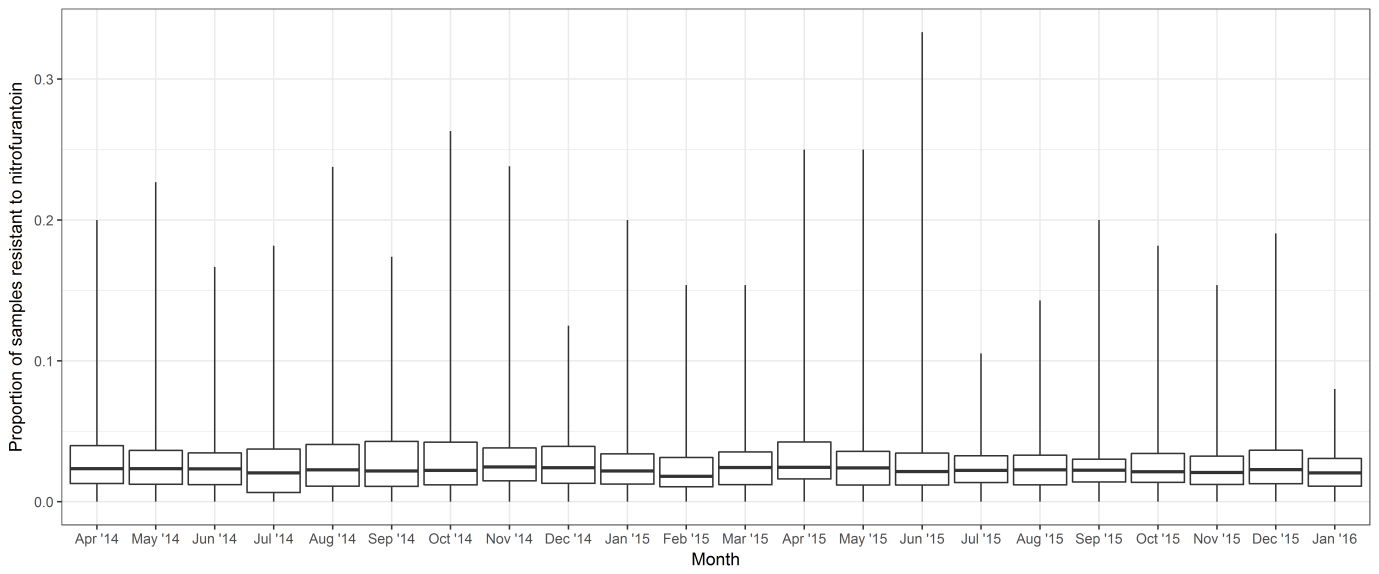


**Fig G. Proportion of urinary samples with E. coli isolated resistant to nitrofurantoin.**

The boxplot shows the variation in nitrofurantoin resistance between Clinical Commissioning Groups over time.
